# Comparative Genomics Reveals Adaptation by *Alteromonas* sp. SN2 to Marine Tidal-Flat Conditions: Cold Tolerance and Aromatic Hydrocarbon Metabolism

**DOI:** 10.1371/journal.pone.0035784

**Published:** 2012-04-26

**Authors:** Renukaradhya K. Math, Hyun Mi Jin, Jeong Myeong Kim, Yoonsoo Hahn, Woojun Park, Eugene L. Madsen, Che Ok Jeon

**Affiliations:** 1 School of Biological Sciences, Research Center for Biomolecules and Biosystems, Chung-Ang University, Seoul, Republic of Korea; 2 Division of Environmental Science and Ecological Engineering, Korea University, Seoul, Republic of Korea; 3 Department of Microbiology, Cornell University, Ithaca, New York, United States of America; Cinvestav, Mexico

## Abstract

*Alteromonas* species are globally distributed copiotrophic bacteria in marine habitats. Among these, sea-tidal flats are distinctive: undergoing seasonal temperature and oxygen-tension changes, plus periodic exposure to petroleum hydrocarbons. Strain SN2 of the genus *Alteromonas* was isolated from hydrocarbon-contaminated sea-tidal flat sediment and has been shown to metabolize aromatic hydrocarbons there. Strain SN2's genomic features were analyzed bioinformatically and compared to those of *Alteromonas macleodii* ecotypes: AltDE and ATCC 27126. Strain SN2's genome differs from that of the other two strains in: size, average nucleotide identity value, tRNA genes, noncoding RNAs, dioxygenase gene content, signal transduction genes, and the degree to which genes collected during the Global Ocean Sampling project are represented. Patterns in genetic characteristics (e.g., GC content, GC skew, Karlin signature, CRISPR gene homology) indicate that strain SN2's genome architecture has been altered via horizontal gene transfer (HGT). Experiments proved that strain SN2 was far more cold tolerant, especially at 5°C, than the other two strains. Consistent with the HGT hypothesis, a total of 15 genomic islands in strain SN2 likely confer ecological fitness traits (especially membrane transport, aromatic hydrocarbon metabolism, and fatty acid biosynthesis) specific to the adaptation of strain SN2 to its seasonally cold sea-tidal flat habitat.

## Introduction

The genus *Alteromonas*, established by Baumann *et al*. [1], [2], accommodates Gram-negative, aerobic, motile, rod-shaped bacteria living in marine habitats. Members of the genus *Alteromonas* are globally distributed and have been isolated as different ecotypes from surface, as well as deep (1000∼3500 m), seawater [3], [4], [5], [6], [7]. Previous studies also have shown that members of the genus *Alteromonas* may dominate heterotrophic blooms; thus, this genus has been generally described as having a copiotrophic way of life as an *r*-strategist [8], [9]; they can grow rapidly when organic nutrients are available in the marine setting.

The coasts of the Yellow Sea on the Korean peninsula consist of vast tidal flats, which are known as “getbol” in Korea. It is well known that sea-tidal flats are rich in valuable biological resources (including marine animals) that play very important roles in the restoration and stabilization of coastal ecosystems and nutrient cycling [10], [11], [12], [13]. Sea-tidal flats are coastal marshes or muddy areas that undergo flooding with seawater and exposure to the atmosphere between low and high tides [14]. Petroleum hydrocarbon releases into the marine environment occur broadly [15]; in fact, in 2007 the oil tanker MV Hebei Spirit released an estimated 12,547,000 liters (10,900 M/T) of crude oil to tidal flats along the Taean coast of the Yellow Sea in South Korea [16]. During the winter season, microbial communities in sea-tidal flats experience very low temperature (<0°C) especially low tide; while in summer, the sea-tidal flat microorganisms confront the challenge of higher temperature (>30°C). Obviously then, sea-tidal flat habitats present selective pressures for native marine microorganisms (temperature, fluctuations in oxygen tension and organic compounds; Kim et al. [17], [13]) that differ considerably from those of the open ocean.

Previously published reports have suggested that many *Alteromonas*-related bacteria likely play important roles in PAH degradation in marine habitats [18], [19]. Our previous studies also showed that members of *Alteromonas* were responsible for the *in situ* biodegradation of polycyclic aromatic hydrocarbons (PAHs) in crude oil-contaminated sea-tidal flat sediment [20]. Recently, *Alteromonas* sp. strain SN2 (KACC 91504P) was isolated and its genome was sequenced [21]. However, the genetic traits of strain SN2 that confer abilities to metabolize aromatic hydrocarbons and facilitate successful adaptation to sea-tidal flat conditions remain to be explored.

Over the past few years, genomic analysis and comparative genomics have provided significant insights into the ecological fitness traits and metabolic versatilities of microbes. Here, we present an analysis of the whole genome sequence of strain SN2 and compare it with the genomes of two *Alteromonas macleodii* strains representing distinctive ecotypes: surface waters in the Pacific Ocean and deep waters of the Adriatic Sea [7]. We also examine the SN2 genome for special features such as genomic islands, codon usage, membrane transport, signal transduction genes, tRNA diversity, recombination, carbon metabolism, and aromatic hydrocarbon biodegradation–all of which reveal ecological fitness traits associated with microbial survival strategies (especially cold adaptation) that are relevant to sea-tidal flat sediments.

## Materials and Methods

### Sampling site and bacterial strains

*Alteromonas* sp. SN2 (KACC 91504P) was isolated from a sea-tidal flat sediment gathered from the Taean coastal area (36°48′ 50″N 126°11′9″E), Republic of Korea, on December 29, 2007, 22 days after an oil spill accident [20]. At the sampling time, the characteristics of the sampling site were as follows: temperature of the sampling site, 2.0°C; temperature of seawater, 6.7°C; salinity of seawater: 32.05‰; and dissolved oxygen of seawater: 10.9 mg l^−1^. In the previous report, the entire genomic sequence of strain SN2 was determined [21]. In brief, a draft of assembly with the pyrosequencing data at 130×coverage was first generated and all intrascaffold and interscaffold gaps were closed by primer walking and PCR segment sequencing. The final whole-genome sequence was further validated by the Illumina sequencing data (about 150×coverage) and thirty-seven ambiguous regions were finally confirmed by Sanger sequencing [21]. *Alteromonas* sp. AltDE (DSM 17117) and *Alteromonas macleodii* ATCC 27126 which were isolated from the depths (1,000 m, 12.5°C) of the Adriatic Sea [7] and from surface seawater of the Oahu coast (Hawaii) [1], respectively, were used as reference strains for phenotypic and genomic comparisons. For the growth tests for the three strains at different temperatures, cells of the three strains at 30°C overnight were inoculated (1%, vol/vol) into marine broth and cultivated in a shaking incubator at 180 rpm. Growth was monitored by measuring the OD_600_ of the cultures.

### Genome annotation and comparative genomics

**Figure 1:**
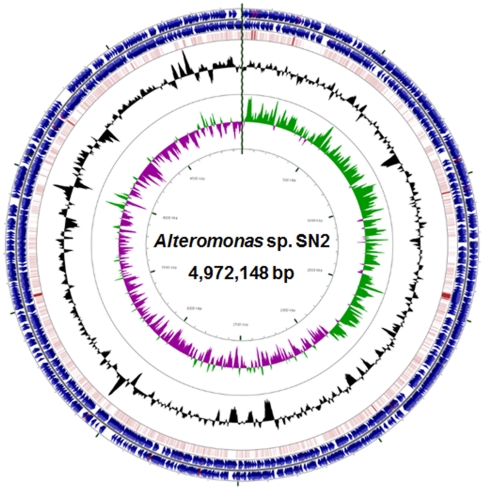
A circular map representing the genome of *Alteromonas* sp. SN2. Forward strand and reverse strand CDSs (blue) are depicted on the outermost two circles of the map, respectively, and RNA genes (tRNA: red; rRNA: violet; others: gray) are also shown on the same circles. The third circle represents the BLASTN comparison of the strain AltDE genome against the strain SN2 genome (dark red indicates highly homologous CDSs). G+C content (black) and GC skews (GC skew+: green, GC skew-: violet) are drawn on the fourth and fifth circles, respectively.

**Figure 2:**
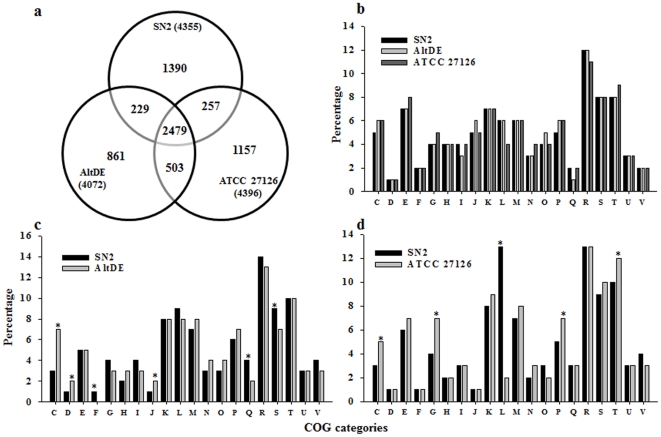
Comparison of the gene content of strains SN2, AltDE, and ATCC 27126. (a) A Venn diagram of shared and specific CDS genes in each strain. Percentages of COG categories in the three *Alteromonas* species. (b) All orthologous genes and (c) specific orthologous genes between strains SN2 and AltDE and (d) between strains SN2 and ATCC 27126. The alphabetic code for the column charts is as follows: C, energy production and conversion; D, cell division and chromosome partitioning; E, amino acid transport and metabolism; F, nucleotide transport and metabolism; G, carbohydrate transport and metabolism; H, coenzyme metabolism; I, lipid metabolism; J, translation, ribosomal structure, and biogenesis; K, transcription; L, DNA replication, recombination, and repair; M, cell envelope biogenesis, outer membrane; N, cell motility and secretion; O, posttranslational modification, protein turnover, and chaperones; P, inorganic ion transport and metabolism; Q, secondary metabolite biosynthesis, transport, and catabolism; R, general function prediction only; S, function unknown; T, signal transduction mechanisms; U, intracellular trafficking, secretion, and vesicular transport; V, defense mechanisms. Asterisks appear when a difference between treatments was at least 20%.

The completed genomic sequence of strain SN2 was submitted to JGI Integrated Microbial Genomes (IMG, http://img.jgi.doe.gov/) for automatic annotation; the results are available at URL https://merced.jgi-psf.org/cgi-bin/er/main.cgi. Genomic sequencing and other related information for strains AltDE and ATCC 27126 were obtained from the IMG server. A circular map representing the genome of strain SN2 in Figure 1 was generated using the web-based CGview program [22]. Shared proteins in Figure 2(a) were defined as the reciprocal best-hit proteins with a minimum of 50% identity and to 70% of the length of either protein, as calculated by the BLAST algorithm. Proteins with no matches were considered to be strain-specific proteins. The COG analysis illustrated in Figures 2(b), 2(c), and 2(d) was performed with the Function Category comparisons tool at IMG. Number and diversity of tRNA genes of Table 1 were retrieved from the tRNA database (http://gtrnadb.ucsc.edu/; [23]) or were calculated using an online web service (http://mobyle.pasteur.fr). The ANI (average nucleotide identity) was calculated using the JSpecies web program [24]. Small non-coding RNAs (ncRNA) were identified by the IMG server and their roles were predicted using the web-based TargetRNA program ([25]; http://snowwhite.wellesley.edu/targetRNA/). Transporters, insertion sequence (IS) elements, transcriptional regulators, chaperones and other functional genes mentioned in Table 2 were found by inputting those terms in NCBI or IMG gene product searches. Chromosomal synteny of strains SN2 and AltDE was viewed using the tool within the IMG server, and their genomic comparison was also performed using the Mauve program [26]. Karlin signature skew and cumulative GC skew were retrieved using Artemis tools (sact_v9.0.5) [27]. The tetranucleotide frequency skew was drawn using the Oligoweb interface (http://insilico.ehu.es/oligoweb/). The Island Viewer was used to identify chromosomal deviation in GC content, so called Genomic Islands (GIs) (http://www.pathogenomics.sfu.ca/islandviewer; [28]). The CRISPR gene sequences in the strain SN2 genome were found using an online web service (http://crispr.u-psud.fr/Server/CRISPRfinder.php). CUSP and CODCMP from the European Molecular Biology Open Software Suite (EMBOSS) package [29] were used for codon usage deviation analysis of the genomic islands (GIs), as shown in Table 3. Correspondence analysis of codon usage as shown in Table 4 was carried out using the web-based codonw 1.4.4 program (http://mobyle.pasteur.fr).

**Table 1:** General features of the whole genomes of three *Alteromonas* strains^a^.

	SN2	AltDE	ATCC 27126
Size (bp)	4,972,148	4,412,285	4,607,010
GC content	43.51	44.90	44.60
Contigs	1	1	716
Total genes^b^	4,442	4,128	4,449
Protein coding genes	4,355	4,072	4,396
Proteins with function prediction	3,401	3,032	3,306
DNA coding density (%)	87.99	86.65	86.76
Proteins assigned to COG (%)	3,089 (70.93)	2,864 (70.33)	3,134 (71.29)
Average gene length (bp)	999.18	933.97	909.33
rRNA operons	5	5	5
Total tRNA genes	64	40	48
tRNA diversity	34	29	31
ANI^c^ (%)	-	74.03	70.80
^Non-coding RNA^	8	-	-

a The genome analysis was carried out at JGI Integrated Microbial Genomes (http://img.jgi.doe.gov/).

b Numbers of total protein coding genes.

c ANI, Average nucleotide identity [34].

**Table 2:** Abundances of gene categories found in the three genomes of *Alteromonas* strains SN2, AltDE, and ATCC 27126.

Genes categories	SN2	AltDE	ATCC 27126
Phage integrase	13	32	12
Transposases and IS elements	66	65	3
Chaperones	17	13	9
Sigma factors	18	8	10
Dioxygenases	21	11	8
TonB receptor	60	52	82
ABC transporters	73	82	79
Heavy metal resistance	14	36	24
Toxin-antitoxin system	3	12	7
Acr system	51	43	50
Histidine kinases	48	40	53
DDGEF domain proteins	24	24	22
Diguanylate cyclase	15	6	14
Antioxidation-related genes	23	17	15

**Table 3:** Characteristics of genomic islands (GIs) found in *Alteromonas* sp. SN2.

GI	Size (kbp)	Number of genes	GC content (%)	Hypothetical proteins	Number of transposase and integrase genes	Predicted function	Codon usage deviation^a^
1	12.82	12	0.42	7	2	Membrane transport	2.089
2	15.30	16	0.40	11	3	Unknown	2.859
3	7.19	8	0.42	6	0	Cytochrome complex	2.423
4	6.81	7	0.40	3	2	Cysteine synthesis	3.547
5	6.43	11	0.40	9	1	Unknown	2.595
6	9.89	7	0.41	3	2	Fatty acid biosynthesis	2.348
7	10.30	8	0.40	6	0	Recombinase	2.411
8	7.32	7	0.40	4	1	Recombinase	2.476
9	37.50	35	0.39	19	5	Defense/motility	2.482
10	28.58	43	0.48	32	2	Conjugation	2.627
11	63.88	60	0.42	19	4	PAH degradation	1.530
12	4.03	7	0.45	5	0	DNA repair	4.243
13	18.81	17	0.49	9	1	Energy production or conversion	2.915
14	8.81	12	0.43	5	5	LOS biosynthesis	2.193
15	18.47	15	0.39	6	3	GreB transcriptional factor	2.514

Abbreviations: PAH, polycyclic aromatic hydrocarbons; LOS, lipooligosaccharide.

a Sum of the differences in codon use for each nucleotide triplet between a particular genomic island and the whole genome.

**Table 4:** Correspondence analysis of codon usage among three *Alteromonas* species genomes.

Organism	T3	C3	A3	G3	GC	GC3s	Fop	CBI	CAI	L_sym	L_nsym
SN2	0.3521	0.2776	0.3433	0.2647	0.450	0.429	0.409	−0.004	0.187	1509464	53617
AltDE	0.3403	0.2820	0.3320	0.2759	0.463	0.445	0.416	0.009	0.188	1346658	45921
ATCC 27126	0.3396	0.2788	0.3349	0.2763	0.460	0.443	0.415	0.008	0.191	1409307	47524

Eleven types of codon use are shown for *Alteromonas* strains SN2, AltDE, and ATCC 27126

Abbreviations: T3,C3,A3 and G3 indicate the frequencies of bases at position 3 of each codon.

GC, GC content in coding genes (G+C)

GC3s, GC of silent 3^rd^ codon position

Fop, frequency of optimal codon index

CBI, codon bias index

CAI, codon adaptation index

L_sym, number of synonymous codons

L_nsym, number of non-synonymous codons

### Global ocean sampling (GOS) recruitment

**Figure 3:**
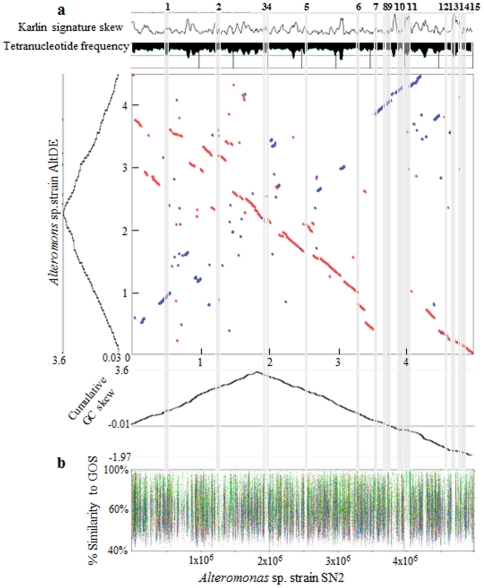
Genome-wide analyses of *Alteromonas* strain SN2. (a) Dotplot showing chromosomal synteny between strains SN2 and AltDE. The blue and orange-colored circles indicate reciprocal best hits in the forward and reverse strands for amino acid regions with >74% identity. The dotplot shows extensive rearrangement in strain SN2 in the form of reciprocal inversions. Plots of cumulative GC skew for strains SN2 and AltDE are shown next to the axes. The Karlin signature difference and tetranucleotide frequency diagrams are shown above the dotplot. Fifteen genomic islands (shaded regions 1–15) were found in the strain SN2 genome. (b) Recruitment of nucleotide-sequence fragments from Global Ocean Survey (GOS) database sequences against the strain SN2 genome using the Mummer program. Geographic origins of the GOS data sets are shown by color codes as follows: Sargasso Sea (green), North American east coast (pink), Coccus Kelling inside London (light green), Galapagos Island (red), Caribbean Sea (light blue), eastern tropical Pacific (brown), Panama Canal (yellow), Indian Ocean (sky blue), tropical South Pacific (dark green), Polynesian Archipelagos (dark blue).

Recruitment of nucleotide-sequence fragments from GOS database sequences [30], [31] by the three *Alteromonas* genomes were performed as described by Ivars-Martinez et al., [7]. Prior to performing the recruitment analysis, the data base was sorted according to the temperature (34 data sets spanning source-water temperatures from 2 to 37 °C). The analyses used BLASTN set for a cutoff of 50% identity and 70% of the length of the query sequence. Normalization of the results was performed based on database size, and the distribution of BLASTN best hit values of SN2 (along with the values of strains AltDE and ATCC 27126) were plotted using the SigmaPlot program (Systat Software, USA). Recruitment of GOS database sequences to the strain SN2 genome, as shown in Figure 3(b), was performed using the MUMmer program [32] and was visualized using the plotting program gnuplot (http://www.gnuplot.info/) after sorting subsets of the GOS database by the following geographic origins: follows, Sargasso Sea, North American east coast, Coccus Kelling inside London, Galapagos Island, Caribbean Sea, eastern tropical Pacific, Panama Canal, Indian Ocean, tropical South Pacific, Polynesian Archipelagos.

### Oxidative stress sensitivity assay and growth curves

Growth (OD) by all three strains was monitored in marine broth (Difco, USA) at different temperatures (5 to 40°C). Assays were performed in triplicate in 5-ml culture volumes, shaken a 120 rpm, and periodically monitored with a spectrophotometer. Oxidative stress sensitivities of the three *Alteromonas* strains (SN2, AltDE, and ATCC 21726) were evaluated as previously described [33]. Cells were grown in marine broth overnight at 30°C and diluted 10-10^4^-fold in the same medium. The diluted cells were spotted on marine agar (MA) without or with H_2_O_2_ (700 µM) and incubated at 30°C for 24 h.

### Accession number

The complete genome sequence of strain SN2 has been deposited in GenBank under accession no. CP002339.

## Results and Discussion

### General features of the strain SN2 genome

Strain SN2 has a single circular chromosome of 4,972,148 bp with a G+C content of 43.51% [17]. General aspects of the genome of strain SN2 and the two *Alteromonas macleodii* strains, AltDE and ATCC 21726, are summarized in Table 1. Key structural features, including G+C content, GC skew, and coding sequences (CDSs) shared with strain AltDE (all discussed below) are graphically depicted in Figure 1. Strain SN2 has a larger genome (4.97 Mb) than the two other *Alteromonas* strains, AltDE (4.41 Mb) and ATCC 27126 (4.61 Mb). The average gene length of strain SN2 was relatively longer than those of the other two *Alteromonas* strains because the number of protein coding genes in strain SN2 did not exceed that of strain ATCC 27126, and the DNA coding density of strain SN2 (87.99%) was similar to that of strains AltDE (86.65%) and ATCC 27126 (86.76%) (Table 1). The genome of strain SN2 encodes 4,355 candidate protein coding genes, of which 3089 (70.93%) proteins were assigned to the cluster of orthologous groups of proteins (COG), similar to the values for strains AltDE (70.33%) and ATCC 27126 (71.29%).

Comparative average nucleotide identity (ANI) analysis with all sequenced bacterial genomes in the IMG database revealed that the genome of strain SN2 is most closely related to strains ATCC 21726 and AltDE. The ANI values between strains SN2 and AltDE and ATCC 21726 were 74.03% and 70.8%, respectively, and the ANI value between strains AltDE and ATCC 27126 was 81.24% [7], which indicates that, contrary to existing nomenclature, the three *Alteromonas* strains represent members of different species within the genus *Alteromonas* [34]. Five complete sets of rRNA genes, 64 tRNA genes, and eight non-coding RNA genes were identified within the strain SN2 genome.

### Comparing gene contents across genomes

Distinctive genetic traits of strain SN2 may provide valuable clues in identifying selective pressures and evolutionary developments that have allowed strain SN2 to succeed in its tidal flat sediment habitat. Therefore, we applied established criteria (minimum percent identity of nucleotides, 50%; coverage, 70%) to identify common and strain-specific genes among the three *Alteromonas* genomes (Figure 2). The three genomes shared 2479 core protein coding genes [Figure 2(a)]. Strains AltDE and ATCC 27126 shared more genes with each other than with strain SN2 [Figure 2(a)]. This implies that the SN2 genome contains a greater proportion of strain-specific genes than the other two genomes– likely encoding specialized physiological properties. Although a COG analysis comparison using the total pool of genes reinforced commonalities among the three genomes (Figure 2b), contrasts found using strain-specific genes appeared more evident, especially in the COG categories of energy production and conversion (C), carbohydrate transport and metabolism (G), and replication, recombination and repair (L) [Figures 2(c) and 2(d)].

Genes from the categories of greatest contrast in Figures 2(c) and 2(d) were subjected to additional scrutiny (Table 2). Strain SN2's genome contains 66 transposase and IS elements belonging to 16 families; this abundance of mobile genetic element-related genes is similar to that of the genome of strain AltDE (65) but is much higher than that of the genome of strain ATCC 27126 (3) (Table 2). Regarding another type of mobile genetic element, the genomes of strain SN2 and ATCC 27126 both contain a relatively low number (13 and 12, respectively) of phage integrase genes, compared to that of the AltDE genome (32). The current literature exhibits conflicting trends regarding the influence of ocean-water depth on the abundance of mobile genetic elements. Reports indicate that metagenomic DNA from surface seawater can contain fewer phage integrases than that of deep (4000 m) seawater [7], [35]. On the other hand, DeLong et al. [36] observed that phage genes were enriched in the photic zones having high productivity. We feel the trends of phage genes found within our three compared genomes reflect a combination of both water depth and the sediment matrix. We speculate that microorganisms living in sea-tidal-flat sediment experience cell-cell interactions at relatively high frequency (leading to lateral gene transfer) due to relatively high cell densities associated with particulate surfaces [35]. By contrast, exposure in sediment to attack by phage parasites may be relatively infrequent due to adsorptive inactivation of the phage by particle surfaces [7].

Strain SN2 harbors more dioxygenase genes than the other two *Alteromonas* strains AltDE and ATCC 27126 (Table 2), indicating that strain SN2 has a higher degradation potential for recalcitrant organic compounds such as polycyclic aromatic hydrocarbons (PAHs). However, TonB receptors, which are related to the transport or utilization of various substrates, are not as prevalent in strain SN2 as they are in ATCC 27126. Similarly, there are comparatively fewer ABC transporter genes in strain SN2 than in the other two strains (Table 2), which contrasts with a previous report that prokaryotic species with larger genome may contain more ABC transporter genes [37]. Both trends in transport-related genes may reflect the likelihood that carbon flow in sea-tidal flat habitats may be proportionately less reliant on photosynthesis than is surface seawater and less reliant on organic particulates (e.g., marine snow) than are deep sea areas. Strain SN2 contains fewer heavy metal resistance-related genes and toxin-antitoxin systems. Phenotypically, however, our recent experimental results (Figure S1 and Table S1) showed that resistance by strain SN2 to heavy metals (zinc and mercury; also antibiotics) was similar to that of strain AltDE [7]. Genes that enable export of drugs or toxins are commonly found in Gram-negative bacteria. The ability of these three *Alteromonas* strains to tolerate exposure to metals and antibiotics may be facilitated by their substantial content (SN2, 51; AltDE, 50; ATCC 27126, 45) of Acr system-related genes.

Although most genes in strains SN2 and AltDE matched significantly, the complex patterns exhibited in the chromosomal synteny dot-blot revealed that numerous genomic translocation, inversion, and insertion have occurred (Figure 3; the dot-blot plot between strains SN2 and ATCC 27126 was not shown due to the incomplete genome sequence of strain ATCC 27126); such rearrangements may have been mediated by strain SN2's mobile genetic elements (transposases, IS elements, phage integrases; see above and Table 2). A global genomic comparison using the Mauve program also confirmed the extensive genome-wide rearrangements in strain SN2 relative to strain AltDE, especially in the form of reciprocal inversions (Figure S2). Furthermore, deviations from mean values in Karlin signature skew, cumulative GC skew, and tetranucleotide frequency (Figure 3) show significant correlation; thereby providing additional evidence for structural rearrangements in strain SN2's genome. Interestingly, the cumulative GC skew in the genome of strain SN2 showed asymmetry (Figure 3); this is fully consistent with the above-suggested recent genetic acquisitions and rearrangements [38]. Fifteen genomic islands were detected across the SN2 chromosome; these are discussed below.

### Testing temperature adaptation among three *Alteromonas* genomes using gene-fragment recruitment analysis of the GOS database

**Figure 4:**
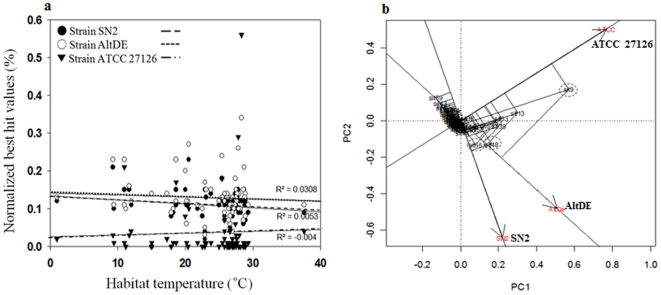
Recruitment analysis of the GOS database sequences sorted by source-water temperatures. (a) Distribution of the normalized BLASTN best hit values recruited against the three marine *Alteromonas* genomes as a function of habitat temperature. (b) Principal Components Analysis (PCA) plot of data shown in panel A, showing results of factor analysis, and sorting by matched genome.

Gene-fragment “recruitment analysis” has recently proven to be an effective tool for assessing the degree of representation of reference genomes in environmental metagenomic sequence reads. By mapping the metagenomic reads onto known genomes, this approach has shown that *Pelagibacter ubique* had a high presence in pyrosequenced fosmid libraries prepared from Norwegian coastal waters [39] and has shown high (>40%) *Alteromonas-*genome coverage in a pyrosequenced metagenome from the Sea of Marmara [40]. Particularly germane to the present investigation prior to carrying out recruitment analyses, Ivars-Martinez et al. [7] sorted subsets from the Global Ocean Survey (GOS) data sets [30,31) according to location of origin and both habitat type (e.g., coastal, estuary, open ocean) and water temperature; this strategy showed that the strain ATCC 27126 genome was highly represented in metagenomes from estuaries and in metagenomes derived from waters >20°C, relative to the AltDE genome. Here we also utilized recruitment analysis to examine the influence of temperature on the degree of representation of each of the three *Alteromonas* genomes. We sorted the GOS database into 82 subsets spanning temperatures from 2 to 37 °C; the majority of the subsets (gathered from depths of 1 to 30 m) were warm surface waters (25 to 29°C). Results of this “recruitment versus water temperature” analysis appear in Figure 4(a), which plots habitat temperature against normalized best-hit values for each of the three *Alteromonas* genomes (a BLASTN cutoff of 50% nucleotide identity was utilized; 70% of the length of the query sequence; best hit numbers were normalized according to database sizes). The recruitment analysis showed that the strain SN2 genome recruited slightly more GOS database genes from seawater samples with low temperature, and vice versa for strain ATCC 27126 although strains SN2 and ATCC 27126 genomes had no significant correlations for source seawater temperature [Figure 4(a)]. A subsequent PCA plot revealed clearer correlations between the seawater temperatures of GOS databases and the normalized best-hit values. In the recruitment analysis of the strain ATCC 27126 genome, the variability represented by PCA factor 1 had a clear positive correlation for the variability represented by factor 2 [Figure 4(b)]. However, in the strain SN2 genome, the two variabilities had negative correlations, which were stronger than those of the strain AltDE genome. Overall, the PCA-analysis showed that the temperature-sorted recruitment trends were distinct for each of the *Alteromonas* genomes. Thus, we conclude that strain SN2 is adapted to grow at low temperature (relative to strains AltDE and ATCC 27126). Conversely, consistent with the findings of Ivars-Martinez et al. [7], we found that strain ATCC 27126 is adapted to grow at relatively higher seawater temperatures.

### Experimental verification of the influence of temperature (5°C to 40°C) on the growth of three *Alteromonas* strains

**Figure 5:**
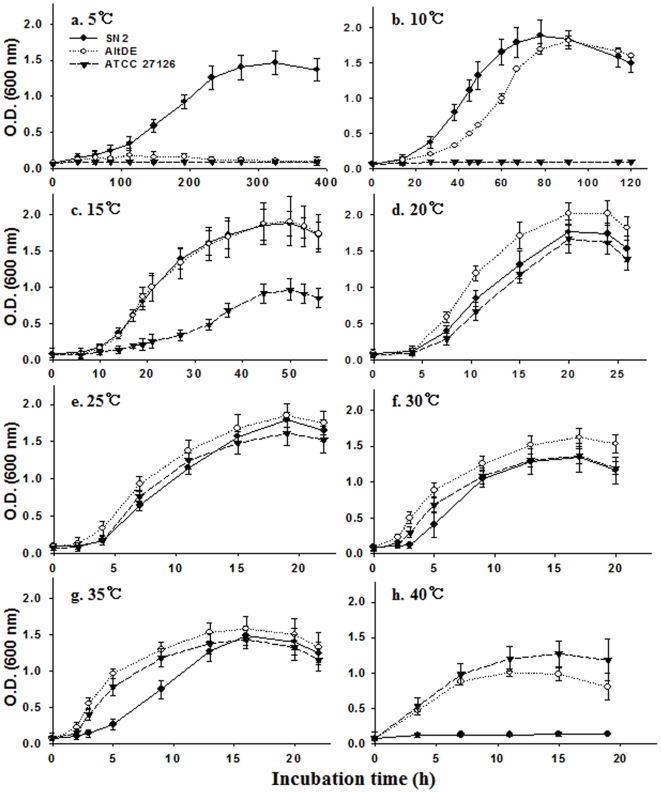
Growth (optical density) of three *Alteromonas* strains (SN2, AltDE and ATCC 27126) in marine broth at eight different temperatures ranging from 5 to 40°C. Closed circles indicate the growth rate for strain SN2, open circles for strain AltDE and closed triangles for strain ATCC 27126. Data show averages of three replicate tubes.

Given the results of the above bioinformatics approach to gain insight into temperature adaptations of the three *Alteromonas* strains, we sought experimental laboratory verification. The sea-tidal flat where strain SN2 was isolated features highly dynamic environmental conditions: coastal waters ebb and flow on lunar cycles, while there are large annual temperature fluctuations (−5°C to 35°C; [13]). By contrast, the seawater habitats where *Alteromonas macleodii* “deep ecotype” (AltDE) and *Alteromonas macleodii* ATCC 27126 were isolated have relatively constant temperatures. AltDE is from the 1,000-m depth of the Adriatic Sea (12.5°C; [7]) and strain ATCC 27126 is from surface seawater off the coast of Oahu (Hawaii), whose annual variation is ∼24 to 28°C. Because of the radically different temperature regimes in the habitats from which the three strains were isolated, we hypothesized their physiologies would reflect this selective influence. Growth (OD) by all three strains was monitored in marine broth at eight different temperatures (5 to 40°C; Figure 5). Strain SN2 clearly grew well at low temperatures– reaching high cell density at 5°C within 14 days, while strains AltDE and ATCC 27126 showed no appreciable growth. At 10°C, both strains SN2 and AltDE showed maximum OD within 80 h, while strain ATCC27126 failed to grow (Figure 5). At 15°C after 50 h, strain ATCC 27126 showed increased OD, but the value was only half that of the other more cold-tolerant strains. At 25, 30, and 35°C growth of the three strains roughly matched one another. Strikingly, at 40°C strain SN2 did not grow appreciably, while the ODs for strains AltDE and ATCC 27126 were diminished, relative to the moderate temperatures (Figure 5). These results clearly confirm that the individual physiologies of the three strains reflect temperature regimes in their habitats of origin: strain SN2 has adapted to cold and ATCC 27126 has adapted to warm.

Psychrotolerant and psychrophilic microorganisms can grow and colonize efficiently even at sub-zero temperatures. Such abilities in microorganisms rely upon a broad array of cellular modifications to counteract low-temperature challenges such as low water viscosity, reduced fluidity of lipid membranes, and low enzymatic activity [41], [42]. Metabolic genes and genomic features of strain SN2 that are likely involved in adaption to the sea-tidal-flat habitat are explored below.

### Genomic islands and other evidence of horizontal gene transfer

Fifteen regions across the SN2 genome were identified (using the integrated mode of IslandViewer) as genomic islands (GIs; shaded regions 1–15 of Figure 3). Table 3 lists characteristics of each GI, including size (kbp), number of genes, number of associated mobile genetic elements, number of hypothetical proteins, and their predicted function. These GIs represent functional gene clusters acquired by strain SN2, likely via relatively recent lateral gene transfer ([43], [44], [45], [46] see above). When fragment recruitment analysis using GOS database sequences was performed for strain SN2's genome [Figure 3(b)], it became evident that some of these GIs were under-represented [particularly GI-5 (unknown function) and GI-13 (energy production/conservation; and also GI-4 (cysteine synthesis), GI-10 (conjugation) and GI-11 (PAH degradation)]. These observations are consistent with the hypothesis that a subset of the genomic islands may have been acquired from bacteria native to terrestrial habitats adjacent to the sea-tidal flat sediment (Table 3). Consistent with this hypothesis is the fact that the majority of GI-1's 7 hypothetical proteins had high similarities to homologues in the genome of the terrestrial soil bacterium, *Pseudomonas mendocina* strain ymp.

Independent of their terrestrial versus marine origins, genomic islands provide additional clues about genome plasticity in strain SN2. Studies of deep-sea microbial genomes have indicated a high degree of genetic plasticity [35], endowed by mobile elements such as phage integrases or transposases (see related discussion, above, and Table 2). GI-10 carries genes encoding TraL, TraE, TraK and a related putative transfer protein (Table 3; loci AMB_00035480, 00035490, 00035500, and 00035510); these contribute to the overall pool of transposase and IS elements impacting genetic rearrangement and horizontal gene transfer. In addition, the IMG database annotation shows that the strain SN2 genome has 37 phage-related genes. Consistent with this, GIs -2, -5 and -9 have footprints of phages or their remnants. Furthermore, GIs -7, -8, -9, and -12 contain DNA-invertase, recombinase, transcriptional regulator, and a restriction modification system, respectively; these all reflect phage-related genetic modifications and selective pressures. Furthermore, both GI-4 (cysteine synthesis) and GI-12 (DNA repair) contain genes that have abnormal codon usage (Table 3), a clear indicator of acquisition through lateral gene transfers [47]. Finally, a survey of COG “L”- category genes from the three *Alteromonas* strains [Figures. 2(b) and 2(c)] revealed that strain SN2 had relatively more genes (221) related to replication, recombination and repair (COG L), while strain AltDE and strain ATCC 27126 had only 178 and 137 genes, respectively.

Thus, the many genomic islands, in combination with mobile genetic elements and DNA replication/repair apparatus present in the genome of strain SN2, offer clear evidence for the prominent role of horizontal gene transfer, phage attacks, and genetic rearrangements in the adaptive evolutionary history of this bacterium.

### Codon usage and correspondence analysis

**Figure 6:**
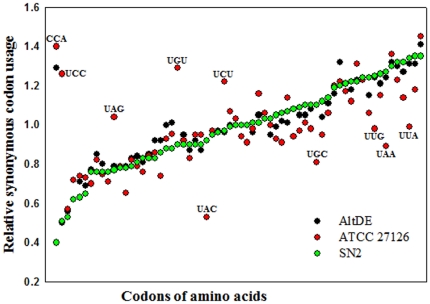
Relative synonymous codon usage (RSCU) in three *Alteromonas* strains (SN2, AltDE, and ATCC 27126). RSCU values were calculated by summing the values for all of the genes. Correspondence analysis of codon usage as shown was carried out using the web-based codonw 1.4.4 program (Rice *et al*., 2000). Codons of amino acids on X axis were arranged based on the ascending RSCU values of strain SN2.

Most amino acids are encoded by more than one nucleotide triplet–there are synonymous codons, which usually differ from one another at the third nucleotide. Such synonymous codons are not used with equal frequencies, and their usage is often distinctive among microorganisms [48]. It is well recognized that natural selection enhances biases of synonymous codon usage; thus, analysis of synonymous codon usage between genomes can facilitate our understanding of the evolution and ecological adaptation [49]. For example, some thermophilic bacteria show very strong triplet biases, including preference for AGG and AUA and strong avoidance of CGU and CGA [50]. We examined relative synonymous codon usage (RSCU) within the genomes of *Alteromonas* strains SN2, AltDE, and ATCC 27126 (Figure 6). While there was broad uniformity in codon usage (Figure 6), strain ATCC 27126 (with its physiological preference for higher temperatures; see above) showed clearly different codon usages of CCA, UCC, UAG, UGU, UAC, UCU, UGC, UUG, UAA, and UUA in comparison to those of the other two *Alteromonas* strains AltDE and SN2. Strains AltDE and SN2 showed very similar codon usage, with the exception of CCA (Figure 6). In search for additional clues regarding low temperature adaptation by strain SN2, we performed correspondence analysis (COA) of codon usage for the three *Alteromonas* genomes; representative COA values for the three *Alteromonas* genomes are listed in Table 4. Although three indices (codon adaptation index, optimal codon index, and effective number of codons) did not reveal major differences (Table 4), strain SN2's genome did show generally lower G and C values for protein-coding genes, and negative value for codon bias index compared to values for the other two *Alteromonas* strains. In particularly, the G and C values of the third nucleotide of synonymous codons in strain SN2 were lower than those of the other two genomes, which is consistent the mounting evidence (see sections above) that strain SN2 is more adapted to cold habitats than are strains AltDE and ATCC 27126 [35].

### Membrane transport and fatty acid biosynthesis: implications for cold-tolerant physiology

It is widely recognized that cold-adapted bacteria feature modified membrane components to maintain cell membrane fluidity at low temperature through the synthesis of unsaturated fatty acids and by changing the lengths of fatty acids and their degree of phosphorylation [51]. Similarly, it is recognized that membrane sterols are very important components in the regulation of membrane fluidity, permeability, and the activity of membrane bound proteins (e.g., transporters) [52], which are common adaptive mechanisms used by cold-adapted microorganisms [53], [54]. Search for sterol-related genes across the strain SN2 genome showed the presence of four sterol desaturase- and one fatty acid desaturase-coding genes, which are in common with two other *Alteromonas* strains. We analyzed the fatty-acid profiles of all three *Alteromonas* strains and also found very few differences (data not shown); we surmise that other cellular factors influencing membrane fluidity may explain the cold adaptation of *s*train SN2. Among four sterol desaturase-coding genes, one (AMB_00013640) was coded on GI-1 and other three (AMB_00002210, AMB_00010260, and AMB_00043730) were coded elsewhere on the chromosome. Strain SN2's sterol-desaturase-containing GI-1 also carries genes encoding a LysR-type transcriptional regulator (AMB_00002200), a maltoporin (LamB, AMB_00002220), and additional putative proteins [Figure S3(a)]. The maltoporin gene, which was only present in strain SN2 among the three *Alteromonas* strains, belongs to the LamB glycoporin family and is predicted to facilitate passage of mono-, di-, and oligosaccharides, nucleic acids, and proteins across the bacterial outer membrane [55].

**Figure 7:**
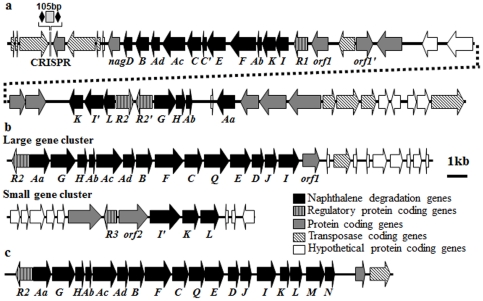
Physical map of a naphthalene degradation gene cluster and transposase coding genes from GI-11 of strain SN2. (a) and its comparisons with *Polaromonas naphthalenivorans* CJ2 (b) and *Ralstonia* sp. U2 (C). The black arrows indicate genes involved in naphthalene metabolism (*nagC*′ is a partial gene of *nagC*). *R1*, *R2*, and *R3* are coding genes of GntR-, LysR-, and MarR-type regulatory proteins, respectively, and *orf1* and *orf2* are coding genes of putative 2-hydroxyhepta-2,4-diene-1,7-dioate isomerase and 3-hydroxybenzoate 6-hydroxylase, respectively.

Strain SN2 also possesses a biotin carboxylase gene (AMB_00042110) in GI-6 that may be involved in *de novo* fatty acid biosynthesis [Figure S3(b); this gene has previously been reported to be expressed in *Sphingopyxis* at low temperature [42]. Strain SN2 carries three other *de novo* fatty acid biosynthesis genes: acetyl-CoA carboxylase (AMB_00010190), cognate biotin carboxyl carrier protein (AMB_00042100), and biotin synthase (AMB_00023740). Except for biotin synthase, other two genes were not found from the genomes of strains AltDE and ATCC 27126. Similarly, biosynthetic genes for the synthesis of cell-membrane aminoglycerophospholipids and the glycerol kinase gene (AMB_00034630; catalyzing phosphorylation of glycerol into glycerol-3-phosphate) are all present in strain SN2, but no glycerol kinase gene was found from other two strains.

In Gram-negative bacteria, membrane components known as lipooligosaccharides (LOS) have been shown to be important for maintaining membrane fluidity at low temperature [51]. The strain SN2 genome harbors clusters of LOS-like genes for the biosynthesis of capsular exopolysaccharides (AMB_00007130,40,50; AMB_00021260,70,80; AMB_00038110; AMB_00033060,70,80); similar gene clusters were also identified in the genome of the relatively cold-adapted bacterium strain AltDE, while such gene clusters were absent from strain ATCC 27126. In addition, lipid A is a component of LOS in Gram-negative bacteria, and lipid A biosynthesis acyltransferase is responsible for lipid A biosynthesis [56]. The lipid A biosynthesis acyltransferase (AMBT_03515) gene was found in GI-14 of strain SN2 [Figure 3S(d)], but was not found in strain AltDE or ATCC 27126.

To conclude, a wide array of genetic characteristics, especially of a maltoporin coding gene, a biotin carboxylase gene, and a variety of other fatty-acid biosynthetic genes, and LOS- related genes likely contribute to the cold adaptation phenotype of strain SN2.

### Signal transduction genes

Histidine kinases constitute the sensing component of the two-component signal transduction systems, and diguanylate cyclase is the GGDEF domain-containing protein that catalyzes the formation of cyclic diguanylate monophosphate (C-di-GMP) [57], [58]. C-di-GMP in bacteria is the ubiquitous secondary messenger involved in bacterial traits such as motility, biofilm formation, phage or metal resistance, virulence, cell-cell communication, and extracellular polysaccharide (EPS) production [57]. A trend has been reported that the genomes of deep seawater bacteria are more enriched in histidine kinases and diguanylate cyclases than are the genomes of surface seawater bacteria [35], [59]. However, our gene-content survey of the three *Alteromonas* strains showed that the deep seawater bacterium, strain AltDE, had far fewer histidine kinases and diguanylate cyclases than did the surface marine habitat strains, SN2 and ATCC 27126 (Table 2). The strain SN2 genome contains 24 genes encoding GGDEF domain-containing proteins, 15 genes encoding diguanylate cyclases, and 48 histidine kinase genes; these match or exceed the corresponding abundances of these two gene categories for the other two strains. Furthermore, strain SN2's genome contains more sigma factor coding genes than those of two other *Alteromonas* strains (Table 2), suggesting that strain SN2 may respond more rapidly to cellular or environmental signals such as temperature fluctuations of the sea-tidal flat. The trend is clear: sea-tidal-flat bacterium strain SN2 harbors more genes specific to signal transduction systems than do its open-ocean-dwelling relatives (strains AltDE and ATCC 27126), suggesting that signal-transduction genes may play proportionately larger roles in the adaptation of strain SN2 to sea-tidal sediment conditions, perhaps fostering surface-attachment by cells to sediment particles.

### tRNAs and non-coding RNAs

The total tRNA gene content in bacteria, especially psychrophilic ones, can contribute to maximum potential growth rates– because high numbers of tRNA genes can compensate for a slow diffusion rates during biosynthetic reactions at low temperatures [55]. Thus, microorganisms with high growth rates (high translational speed and/or accuracy) tend to have a high number of total tRNA genes. It follows that the total tRNA gene content of a genome may be an important indicator of selective pressures at low temperatures [60], [61]. Tallies of tRNA genes across the three *Alteromonas* genomes showed that strain SN2 has a higher total tRNA gene content and tRNA gene diversity than do strains AltDE and ATCC 27126 (Table 1); this observation is, again, consistent with strain SN2's ability to grow well at low temperature compared to strains AltDE and ATCC 27126 (Figure 5).

Another comparative analysis using the IMG server revealed that the genome of strain SN2 carries eight small non-coding RNAs (ncRNAs), which are not present in the other two *Alteromonas* strains (Table 1). Small ncRNAs have been shown to play post-transcriptionally important regulatory roles ranging from Fe^2+^ metabolism to oxidative damage at low temperature to reduction of glucose entry to phosphorylation under various stress conditions [62], [63]. To predict possible roles of the eight ncRNAs in strain SN2, we analyzed the ncRNA sequences using the web-based TargetRNA program [25], which predicts ncRNA interaction genes based on *E. coli* and *Shewanella oneidensis* genomes. Among the eight ncRNAs, the possible regulatory roles of two ncRNAs were predicted in strain SN2. One ncRNA (182 bp) was predicted to bind with mRNA transcripts of *omp*R (*S. oneidensis*) or *usp*A (*E. coli*), suggesting that the ncRNA may regulate the *omp*A (AMB_00039900) gene expression of strain SN2 to improve outer-membrane permeability [34], [64]. The other ncRNA (344 bp) was predicted to bind with mRNA transcripts of GGDEF (*S. oneidensis*) or RpoS (*E. coli*) genes that likely confer tolerance and improve environmental fitness under stress conditions [57]. The above findings suggest that strain SN2's complement of both tRNA and ncRNAs genes add to the bacterium's array of fitness traits.

### Chaperones

Chaperones are ubiquitous and abundant proteins that assist in cell function in all organisms. Chaperones stabilize the conformation and 3-dimensional folding properties of other proteins and have been shown to contribute to bacterial growth at low temperatures [65]. Strain SN2 demonstrated robust growth at 5°C, although its lag period was long (see above, Figure 5). Such phenotypic behavior has often been observed in psychrotrophic and mesophilic bacteria, and is associated with the expression of cold-shock chaperones [65]. As expected, strain SN2 contains more chaperones such as a flagellin-specific chaperonin, heat shock chaperone (Hsp90 & 70), Dna K, J, lipase chaperone, and Cochaperonin (GroEL, GrpE) than do the other two *Alteromonas* strains (Table 2)– which likely signifies the higher adaptability of strain SN2 to environmental fluctuations such as daily sea-tidal flooding, frequent input of contaminants, and wide seasonal temperature changes [66], [7].

### Central metabolism

The genes typical of the complete Embden-Meyerhof-Parnas (EMP) and citric acid cycle pathways are present in all three *Alteromonas* strains. The glucokinase (AMB_00011720) gene, which encodes the enzyme catalyzing the first step of the EMP pathway for strain SN2, is positioned in the genome near the glucose 6-phosphate-1-dehydrogenase gene (AMB_00011690) of the pentose phosphate (PP) pathway. This close gene arrangement and a proximally located *rpiR*-family transcriptional regulator *hexR* (AMB_00011680) indicate co-regulation of the EMP and PP catabolic pathways in strain SN2. However, the genes coding for glucose 6-phosphate isomerase (AMB_00004020; catalyzing the second step in the EMP pathway) and for phosphoglyceraldehyde transaldolase (AMB_00004030; catalyzing the second step in the PP pathway) are distant from the above adjacently located genes. This gene arrangement is also observed in the other two *Alteromonas* strains.

All genes encoding proteins in the Entner-Doudoroff (ED) pathway, which is used by aerobic prokaryotes to transform glucose to pyruvate, are also present in Strain SN2. The two genes coding 6-phosphogluconate dehydratase (AMB_00011710) and 2-keto-3-deoxyphosphogluconate aldolase (AMB_0011730) are adjacent to one another; this arrangement is the same as that in the other two *Alteromonas* strains, as well as in many other strains such as *Polaromonas naphthalenivorans* CJ2, *Polaromonas* JS 666, *Rhodoferax ferrireducens*, and *Acidovorax* JS42 [46]. Meanwhile, the PP pathway can produce a variety crucial metabolic precursors for nucleic acids, aromatic amino acids and NADPH for biosynthesis and several catalytic reactions [46]. All genes required for the PP pathway are present in all three *Alteromonas* strains, and all three *Alteromonas* strains harbor one copy of the gene encoding ribulose-5-phosphate 3-epimerase, which converts ribulose-5-phosphate to xylulose 5-phosphate. However, only strain SN2 shows broader potential metabolic versatility by harboring, in addition, two copies of the ribulose-5-phosphate 4-epimerase gene. Two genes for the glyoxylate pathway within the citric acid cycle, isocitrate lyase (AMB_00007460) and malate synthase (AMB_00007440), are present in all three *Alteromonas* strains. It is well established that many bacteria accumulate glycogen storage bodies during periods of high carbon source availability [59]. Genes for the storage and use of glycogen including glucose-1-phosphate adenylyltransferase (AMB_00020600), glycogen synthase (AMB_00034280), glycogen branching enzyme (AMB_00011770), and glycogen debranching enzyme GlgX (AMB_00011780) are present in all three *Alteromonas* strains. Thus, the presence of central carbon metabolism genes (EMP pathway, citric acid cycle, ED pathway, PP pathway, glycogen storage, and glyoxylate pathway) in all three *Alteromonas* strains is consistent with prior reports of their copiotrophic life style as *r*-strategists [8], [9].

### Degradation of polycyclic aromatic hydrocarbons

Strain SN2 harbors genes for metabolizing polycyclic aromatic hydrocarbons (PAH) within GI-11 [Table 3, Figure 7(a)], while the other two *Alteromonas* strains do not. Gene homology and associated functions indicate that strain SN2 metabolizes naphthalene via the gentisate biochemical pathway, which is encoded by the *nag* operon in other bacteria. GI-11 contains salicylate-5-hydroxylase (*nagGH*) and gentisate 1,2-dioxygenase (*nag I*) genes (Figure 7), which are the key naphthalene metabolic enzymes in the gentisate pathway in *Polaromonas naphthalenivorans* CJ2 [67], [68] and *Ralstonia* sp. U2 [69]. However, the naphthalene catabolic gene order and operon structures among strains SN2, U2, and CJ2 show striking contrasts (Figure 7). The naphthalene catabolic gene cluster in strain U2 occurs as a single linear array of genes, controlled by one LysR-type regulator (*nagR*). In strain CJ2, control is, again, by *nagR*, and the strain U2-like gene organization is retained, although split into large and small gene clusters; [70]. By contrast, the naphthalene catabolic genes of strain SN2 are scattered broadly and are distributed between two clusters across a 33 kbp section of GI-11. Moreover, three regulatory genes (two LysR-type regulators, AMB_00036020; AMB_00036030; one GntR-type regulator, AMB_00035900) are associated with the PAH genes in strain SN2. The presence of additional regulatory genes may be physiologically advantageous to strain SN2; it has been suggested that refined gene regulatory mechanisms allow cells to adjust their metabolism within a challenging range of conditions. The fluctuating conditions such as sporadic input of hydrocarbon contaminants and seasonal temperature change that prevail in strain SN2's sea-tidal-flat habitat may have selected for an increase in gene copy number or alterations in the associated regulatory system [71].

Overall, the naphthalene metabolic pathway carried by strain SN2 likely converts naphthalene to salicylate, then gentisate, and finally fumarate and pyruvate via the gentisate pathway. The PAH degradation capabilities of strain SN2 have been confirmed experimentally; strain SN2 is able to degrade naphthalene, phenanthrene, anthracene, and pyrene [21]. PAH degradation does not seem to be a common feature of the *Alteromonas* strains, although it has been reported that some *Alteromonas* species can degrade PAH compounds [72], [73]. The recruitment of PAH-degrading genes into strain SN2 is presumed to be an important enabling adaptation for the sea-tidal-flat habitat. The absence of napthalene-catabolic genes in the other two *Alteromonas* strains and the presence of transposases at the two ends of the PAH-degrading gene cluster in GI-11 are fully consistent with acquisition in strain SN2 via horizontal gene transfer.

### Oxidative stress tolerance

Mechanisms to combat oxidative stress are required in all microorganisms that carry out aerobic respiration, as well as those that express dioxygenase-type enzymes (discussed above) used in PAH metabolism. Inspection of the strain the SN2 genome revealed that strain SN2 has a greater abundance of both dioxygenase genes (see above) and anti-oxidation related genes than do the two other strains AltDE and ATCC 27126. This tally of genes that neutralize reactive-oxygen-species such as superoxide and hydrogen peroxide (Table 2). Strain SN2 has six predicted catalase genes (AMB_00000560,90,940, AMB_00001910, AMB_00011020,23980), two superoxide dismutase (AMB_00011220; AMB_00025380), and one alkyl hydrogen peroxide reductase (AMB_00013490) gene, which are similar with the corresponding abundances of these three gene categories for other two strains AltDE (5, 2, and 3 genes, respectively) and ATCC 27126 (4, 3, and 3 genes, respectively). On the other hand, six glutaredoxin and eight thioredoxin genes were found from the strain SN2's genome. However, other two *Alteromonas* strains harbor fewer glutaredoxin (AltDE, 4; ATCC27126, 3) and thioredoxin (AltDE, 6; ATCC27126, 6) genes than strain SN2. The presence of oxidative stress tolerance genes may be a strategy adopted by strain SN2 to deal with reactive oxygen species produced by exposure to the atmosphere or the dioxygenase actions. This feature was checked phenotypically and strain SN2 showed more tolerance to 700 µM H_2_O_2_ than did the other *Alteromonas* strains (Figure S4).

### Phage resistance and lateral gene transfer

A survey for genes related to defensive strategies revealed that strain SN2 harbors five restriction endonuclease genes to degrade alien DNAs and protect from viral attack [43]. One of these is encoded within GI-9 (AMB_00033980) [Figure S3(c)], adjacent to an adenine-specific DNA methylase Mod gene (AMB_00033990), which likely contribute to a restriction-modification system to resist phage attack [7]. Because phage that attack bacteria are abundant in seawater habitats, many seawater bacteria may carry CRISPR sequences which function as an anti-phage defense system via RNA-silencing-like mechanism [74], [75]. Four CRISPR sequences were found in the strain SN2's genome via the CRISPR-finder program. Meanwhile, one CRISPR gene and no CRISPR genes were found from strains AltDE and strain ATCC 27126, respectively [7]. Interestingly, one of the CRISPR genes was detected within GI-11 of strain SN2's genome in association with PAH-degrading genes (Figure 7) and its BlastN analysis showed that a related CRISPR gene sequence occur also in the *Shewanella baltica* OS185 genome with high homology. The other two CRISPR gene sequences in strain SN2 were detected, with 100% homology, in the genomes of *Marinomonas* sp. MWYL1 and *Leuconostoc gasicomitatum*. The above patterns in CRISPR and GI occurrence in strain SN2's genome confirm that an extensive and complex array of horizontal gene transfer events have occurred throughout this bacterium's evolutionary history.

In conclusion, comparative genomic analyses of strain SN2 and two other *Alteromonas* strains, AltDE and ATCC 27126, expand our knowledge of the evolution and adaptation of an important marine genus. Numerous independent criteria (e.g., genomic islands, transposons, IS elements, gene clusters with homologs only in taxonomically-distant hosts) establish that strain SN2 has acquired many genes via lateral gene transfer. A large portion of these genetic acquisitions have contributed to strain SN2's successful adaption to cold conditions and to its ability to metabolize PAH compounds in its sea-tidal-flat-sediment habitat that undergoes wide seasonal temperature fluctuations. The completed genome of strain SN2 will allow us to continue to advance understanding of the physiology, evolution, and ecological fitness of this copiotrophic marine bacterium.

## Supporting Information

Figure S1 **Growth curves (Optical Density) of strain SN2 at different concentrations of mercury (a) and Zinc (b).**
(TIF)

Figure S2 **Genomic alignment showing extensive genome-wide rearrangements in strains SN2 and AltDE in the form of reciprocal inversions.** Forty two homologous blocks in the SN2 genome are shown as identically colored regions linked to the AltDE genome. Regions that are inverted relative to strain SN2 are shifted downward in the genome of strain SN2.
(TIF)

Figure S3 **Genomic islands of strain SN2 involved in membrane transport** (a, GI-1), fatty acid biosynthesis (b, GI-6; D, GI-14), and phage resistance (c, GI-9).
(TIF)

Figure S4 **Plate assay to determine the oxidative stress tolerance ability of three** ***Alteromonas*** **species.** The stress tolerance abilities of the strains were tested using 700 µM H_2_O_2_. The serially diluted cells (10 to 10^4^-fold) were spotted on marine agar (MA) without or with H_2_O_2_ (700 µM) and incubated at 30°C for 24 hrs.
(TIF)

Table S1 Antibiotic tolerance for three *Alteromonas* strains (SN2, AltDE, and ATCC 27126). The tests were performed on marine agar at 25°C for 2 days.
(DOCX)
