# Supplementary figures and images for: Comparative Genomics Reveals Adaptation by Alteromonas sp. SN2 to Marine Tidal-Flat Conditions: Cold Tolerance and Aromatic Hydrocarbon Metabolism
Source: PLoS One. 2012 Apr 26;7(4):e35784. doi: 10.1371/journal.pone.0035784 (PMC3338528; doi:10.1371/journal.pone.0035784)

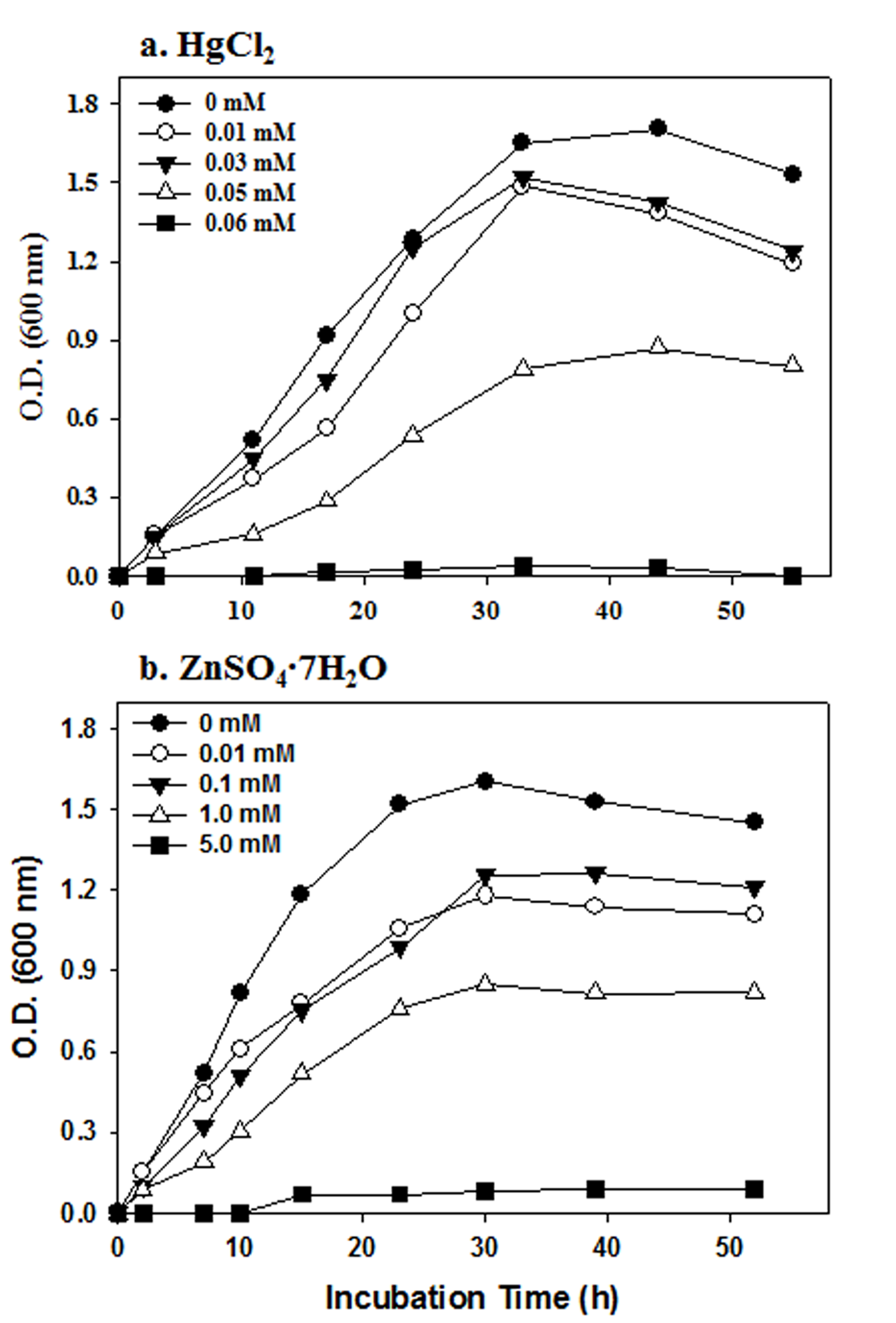

Supplement: Figure S1 — Growth curves (Optical Density) of strain SN2 at different concentrations of mercury (a) and Zinc (b). (TIF) [file pone.0035784.s001.tif]

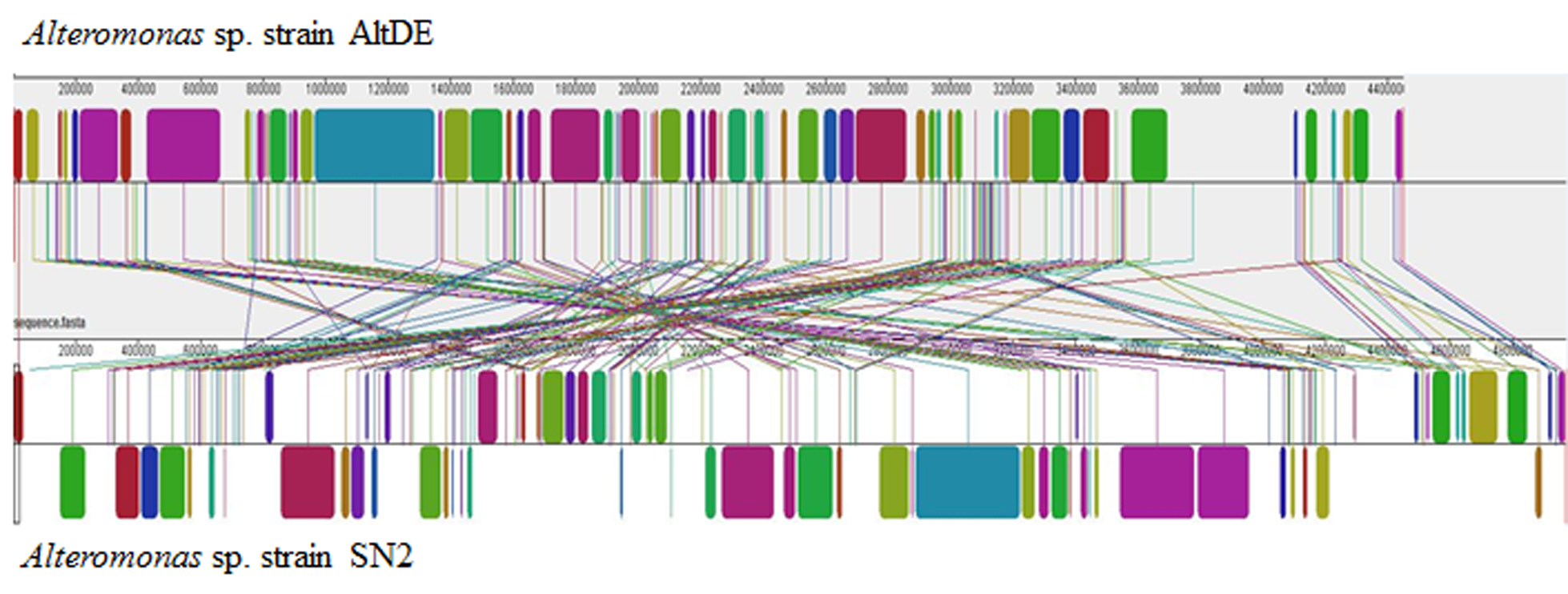

Supplement: Figure S2 — Genomic alignment showing extensive genome-wide rearrangements in strains SN2 and AltDE in the form of reciprocal inversions. Forty two homologous blocks in the SN2 genome are shown as identically colored regions linked to the AltDE genome. Regions that are inverted relative to strain SN2 are shifted downward in the genome of strain SN2. (TIF) [file pone.0035784.s002.tif]

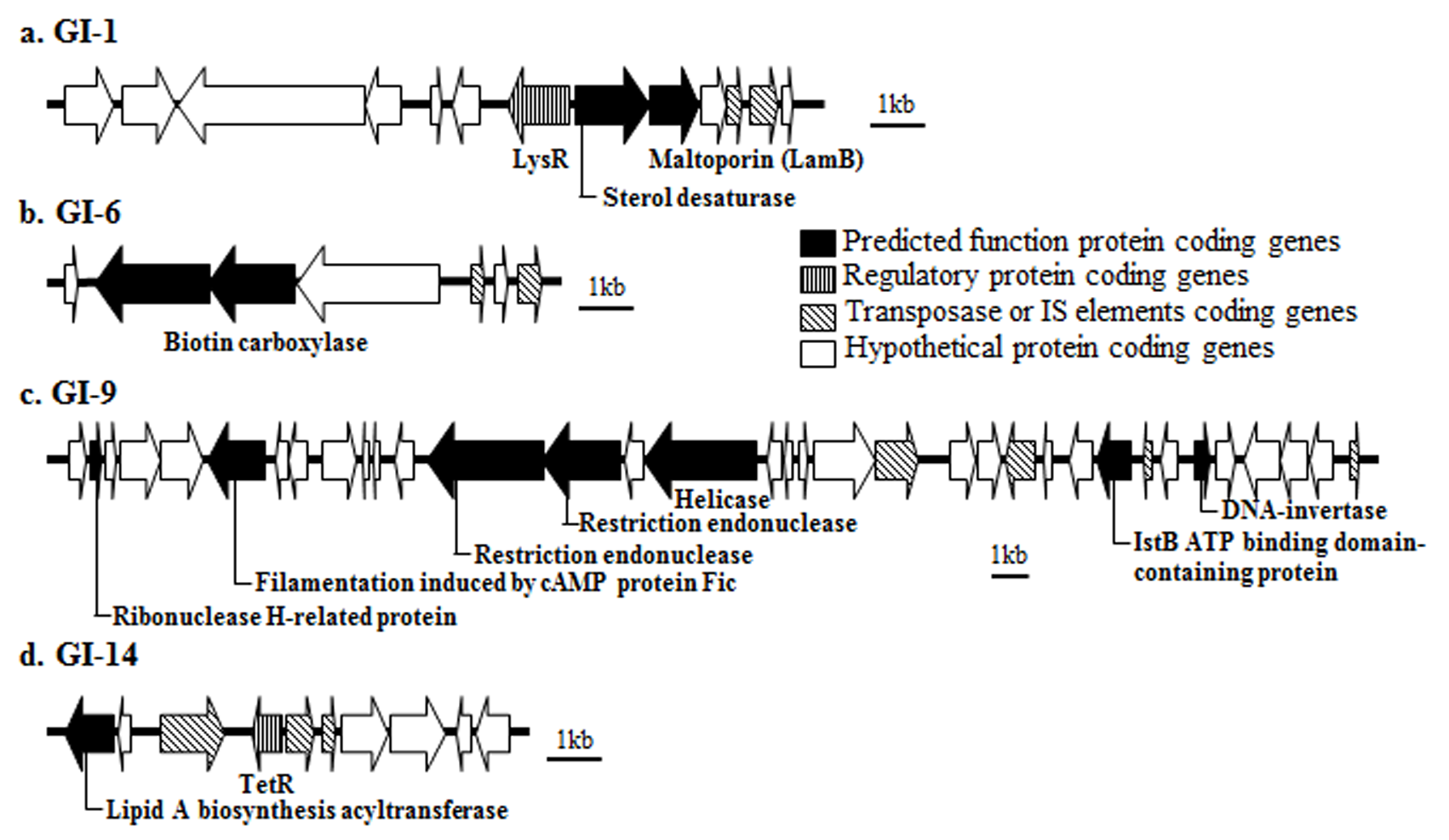

Supplement: Figure S3 — Genomic islands of strain SN2 involved in membrane transport (a, GI-1), fatty acid biosynthesis (b, GI-6; D, GI-14), and phage resistance (c, GI-9). (TIF) [file pone.0035784.s003.tif]

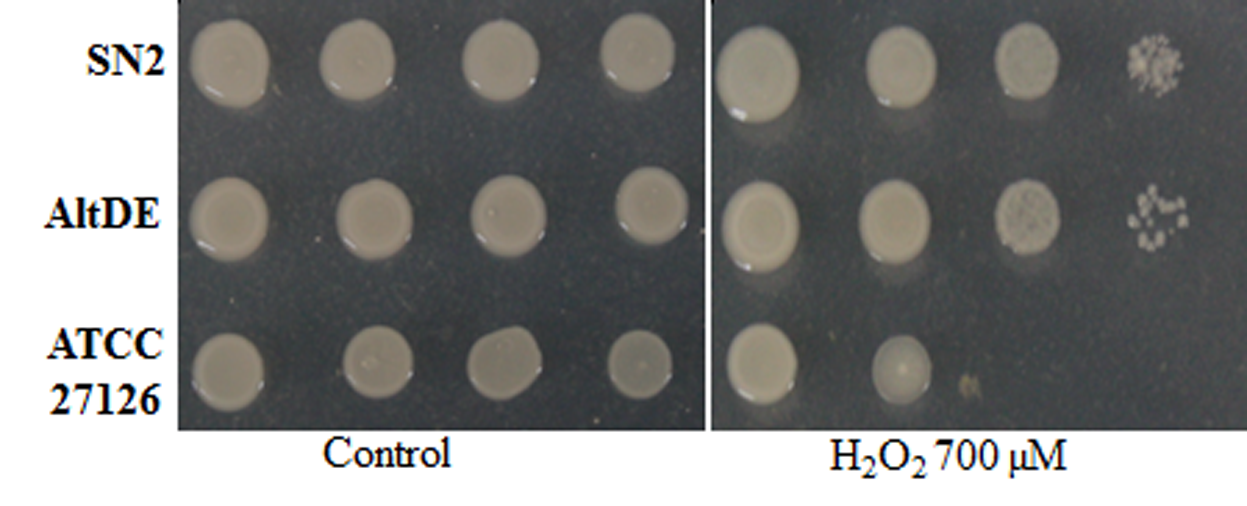

Supplement: Figure S4 — Plate assay to determine the oxidative stress tolerance ability of three Alteromonas species. The stress tolerance abilities of the strains were tested using 700 µM H2O2. The serially diluted cells (10 to 104-fold) were spotted on marine agar (MA) without or with H2O2 (700 µM) and incubated at 30°C for 24 hrs. (TIF) [file pone.0035784.s004.tif]
