# Supplementary material for: Comparative Genomics Reveals Adaptation by Alteromonas sp. SN2 to Marine Tidal-Flat Conditions: Cold Tolerance and Aromatic Hydrocarbon Metabolism
Source: PLoS One. 2012 Apr 26;7(4):e35784. doi: 10.1371/journal.pone.0035784 (PMC3338528; doi:10.1371/journal.pone.0035784)
Supplement: Table S1 — Antibiotic tolerance for three Alteromonas strains (SN2, AltDE, and ATCC 27126). The tests were performed on marine agar at 25°C for 2 days. (DOCX) [file pone.0035784.s005.docx]

**Table S1.** **Antibiotic tolerance for three *Alteromonas* strains (SN2, AltDE, and ATCC 27126). The tests were performed on marine agar at 25°C for 2 days**

| Antibiotic (conc.) | Strains* | | |
| --- | --- | --- | --- |
|  | SN2 | AltDE | ATCC 27126 |
| Kanamycin (10 mg/ml) | - | - | - |
| Oleandomycin (10 mg/ml) | + | + | - |
| Novobiocin (10 mg/ml) | + | + | + |
| Neomycine (100 μg/ml) | - | - | - |
| Tetracycline (5 mg/ml) | + | + | + |
| Lincomycin (10 mg/ml) | + | + | + |
| Ampicilin (50 mg/ml) | + | + | + |
| Streptomycin (50 mg/ml) | - | - | - |
| Polymyxin B (100 U/ml) | - | - | + |
| Chloramphenicol (20 mg/ml) | - | - | - |
| Carbomycin (50 mg/ml) | + | + | - |
| Gentamycin (30 mg/ml) | + | - | + |
| Penicillin (20 IU/ml) | + | + | + |

*+, growth, -, no growth.
